# Sungas Instead of Syngas: Efficient Coproduction of CO and H_2_ with a Single Beam of Sunlight

**DOI:** 10.1002/advs.201500260

**Published:** 2015-10-01

**Authors:** Fang‐Fang Li, Jason Lau, Stuart Licht

**Affiliations:** ^1^ Department of ChemistryGeorge Washington UniversityWashingtonDC20052USA

**Keywords:** carbon monoxide, climate change, hydrogen, solar fuel, syngas

## Abstract

**The electrolytic coproduction of CO and H_2_** is achieved from air, water, and a single beam of sunlight rather than from fossil fuels. H_2_ and CO cosynthesis is driven by a single concentrator photovoltaic to simultaneously drive molten hydroxide and molten carbonate electrolyses. The carbon neutral process captures carbon without the need for the preconcentration of atmospheric carbon dioxide.

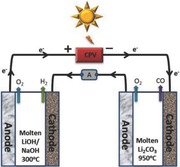

Fossil fuels comprise approximately 80% of global energy sources and are nonrenewable.^1^ When oxidized as fuels, they emit the greenhouse gas CO_2_, which has risen to an atmospheric concentration of 400 ppm during the industrial age and contributes to climate change. Syngas is commonly produced to convert solid‐ or gas‐phase fossil fuels into liquid fuels. In this study, we present the first example of the efficient coproduction of CO and H_2_ syngas with a single beam of sunlight. As summarized in the Supporting Information, steam reformation of methane or coal is commonly used to produce syngas (CO and H_2_); however, the processes release carbon dioxide.^2^ The syngas is then used as a feedstock to form methanol, or for Fischer–Tropsch type reactions, such as

(1) $$n\mathrm{CO}\;+\;(2n\mathrm{+1})H_{2}\to C_{n}H_{\left(2n\mathrm{+2}\right)}\;+\;nH_{2}O$$

Reaction (1) generates the majority of diesel fuel consumed in South Africa, and using coal is less expensive than using oil to produce middle‐distillate range fuels of C11–C18 hydrocarbons, including synthetic jet, kerosene, and diesel fuels.^3^ The disadvantage of that process is the greenhouse gas consequences of the extensive CO_2_ generated in the partial oxidation of coal or natural gas to form CO and hydrogen. This disadvantage is eliminated when hot hydrogen and carbon monoxide are generated instead by sunlight, air, and water.

Nonfossil fuel based, low carbon footprint fuels are needed to ameliorate the effects of anthropogenic climate change. In 2002, we presented a solar electrochemical theory that the full spectrum of sunlight was sufficient to split endothermic electrolysis reactions, such as water to hydrogen fuel at over 50% solar conversion efficiency.^4^, ^5^ In particular, as shown in **Figure** **1**, both the calculated thermochemical water splitting and the carbon dioxide splitting electrolysis potentials to the syngas products (H_2_ and CO, as well as O_2_ at the anode) are endothermic, requiring a smaller potential at higher temperature. The rest potential of common electrolyses, such as *E*(H_2_O → H_2_ + 1/2O_2_) = 1.23 V at room temperature is often considerably greater than the photopotential of visible light semiconductors. Thermal sunlight can provide an efficient heat source to lower the electrolysis potential, opening a pathway to these otherwise potential‐forbidden pathways of charge transfer. This theory was validated by experiment in 2003 showing that even a small bandgap semiconductor, such as silicon, was energetically sufficient to split water when subbandgap sunlight, not used by the semiconductor, was directed for heating the water.^6^

**Figure 1:**
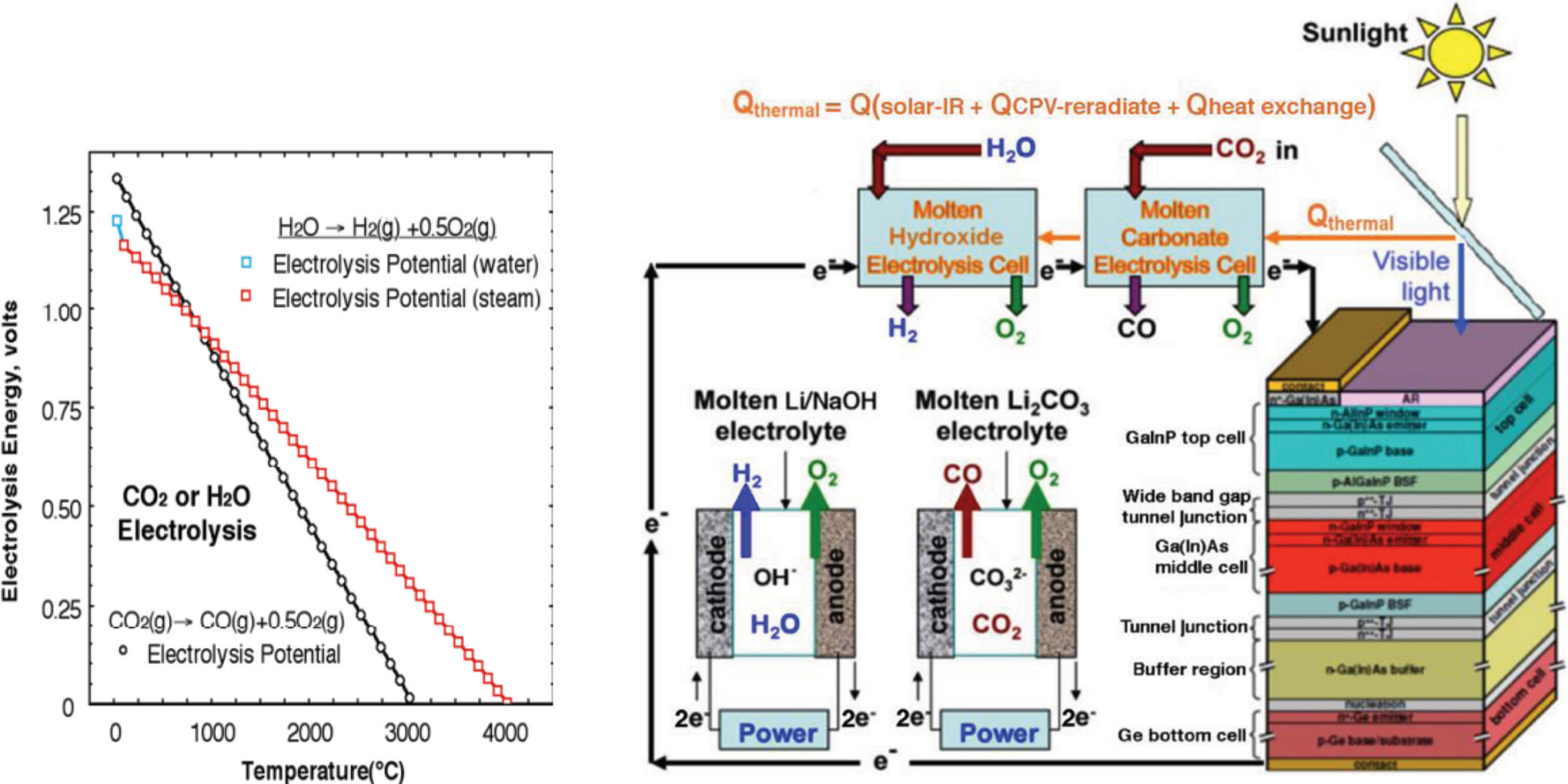
Left: the calculated potential to electrolyze either water or carbon dioxide. The indicated decrease in electrolysis energy, with increase in temperature, provides energy savings in the STEP process in which high temperature is provided by excess solar heat. Energies of electrolysis are calculated with thermochemical values at unit activity using NIST gas and condensed phase Shomate equations.^7^ Right: Schematic of sungas generation. Electronic current from a single CPV drives H_2_ and CO formation through separate molten hydroxide water splitting and molten carbonate carbon dioxide splitting electrolyzers. Electrolyzers are heated by sufficient:^5^, ^6^, ^8^, ^9^ i) excess infrared solar heat (subbandgap), ii) heat reradiated by the CPV preventing the CPV from overheating, and iii) heat exchange between the high temperature formed products and the in‐flowing reactants.

The use of solar thermal energy to lower the potential of useful electrolysis can be applied to liquid, gas, or solid phase electrolyte cells. In general, we have found an energy advantage in applying the solar thermal electrochemical process (STEP) to liquid, molten electrolyte cells. Such cells can be driven by thermal sunlight to high temperature, accommodating a high concentration of reactants, facile kinetics at high current density, and a lower endothermic electrolysis potential. The calculated variations of the water to hydrogen and oxygen, and carbon dioxide to carbon monoxide and oxygen splitting potential are both seen to decrease with increasing temperature in Figure 1.

The Coulombic efficiency of electrolytic water splitting, *η*_H2_ (number of moles of H_2_ generated per 2 F of applied charge), approaches 100% in low melting point and mixed alkali molten hydroxides at temperatures up to 300 °C

(2) $$H_{2}O\to H_{2}+{\mathrm{1/2O}}_{2}$$

(3) $$\mathrm{cathode}:{\mathrm{2H}}_{2}O+{\mathrm{2e}}^{-}\to H_{2}\;+\;{\mathrm{2OH}}^{-}$$

(4) $$\mathrm{anode}:{\mathrm{2OH}}^{-}\to {\mathrm{1/2O}}_{2}+H_{2}O+{\mathrm{2e}}^{-}$$

In 2009, this solar heat facilitated water splitting theory was renamed STEP and generalized to all endothermic reactions.^8^ From 2010, this has been demonstrated for the direct removal of atmospheric or smokestack carbon dioxide (STEP carbon),^9^, ^10^, ^11^ water treatment^12^, ^13^ and organics,^14^ and the CO_2_‐free production of iron (STEP iron),^10^, ^15^, ^16^ magnesium and bleach,^10^ calcium oxide (STEP cement),^17^ and ammonia (STEP fertilizer).^18^

Molten salt cells can often accommodate high reactant concentrations, which lead to a further decrease in the electrolysis potential (as driven by the Nernst equation). For example, a molten carbonate containing dissolved oxide readily absorbs atmospheric CO_2_, and has the advantage that the reducible tetravalent carbon concentration (as carbonate) is 10^6^‐fold higher in concentration than in air (as CO_2_).^8^, ^9^ We have previously demonstrated molten hydroxide electrolytes for solar water splitting to hydrogen fuel,^6^, ^10^, ^19^ and molten carbonate electrolytes for solar carbon dioxide splitting to carbon and carbon monoxide fuels.^9^, ^10^, ^11^ Solid carbon is the observed preferred solar carbon dioxide splitting product at temperature less than approximately 850 °C, while carbon monoxide is the preferred product at temperature greater than 850 °C. At temperatures ≤750 °C, solid carbon is the sole cathode product, and at temperatures ≥950 °C CO is the sole cathode product

(5) $${\mathrm{Li}}_{2}{\mathrm{CO}}_{3}(\mathrm{molten})\to C(\mathrm{solid})\;+\;{\mathrm{Li}}_{2}O(\mathrm{dissolved})\;+\;O_{2}(\mathrm{gas})$$

(6) $${\mathrm{Li}}_{2}{\mathrm{CO}}_{3}(\mathrm{molten})\to \mathrm{CO}(\mathrm{gas})\;+\;{\mathrm{Li}}_{2}O(\mathrm{dissolved})\;+\;{\mathrm{1/2O}}_{2}(\mathrm{gas})$$

When CO_2_ is bubbled in, a rapid reaction back to the original lithium carbonate occurs

(7) $${\mathrm{Li}}_{2}O(\mathrm{dissolved})\;+\;{\mathrm{CO}}_{2}(\mathrm{gas})\to {\mathrm{Li}}_{2}{\mathrm{CO}}_{3}(\mathrm{molten})$$

In the presence of carbon dioxide and dissolved Li_2_O, reaction (7) is exothermic. Lithium oxide is highly soluble in both pure lithium carbonate and the alkali carbonate eutectic, and we observe dissolved Li_2_O decreases the carbonate electrolysis potential.^15^, ^16^ The concentration of lithium oxide can be controlled to maintain the carbonate electrolyte in the CO_2_ absorption mode and eliminate electrolyte carbonate decomposition (the back reaction of Equation (7). The carbon capture reaction in molten carbonate combines Equations (5)–(7)

(8) $${\mathrm{CO}}_{2}(\mathrm{gas})\to C(\mathrm{solid})\;+\;O_{2}(\mathrm{gas});\;\;T\;<850\;{}^{\circ}C$$

(9) $${\mathrm{CO}}_{2}(\mathrm{gas})\to \mathrm{CO}(\mathrm{gas})\;+\;{\mathrm{1/2O}}_{2}(\mathrm{gas});\;T\;\;>\;\;850\;{}^{\circ}C$$

Lubomirsky and co‐workers have also probed the electrolysis of lithium molten carbonates to produce carbon monoxide,^20^ and Chen and co‐workers have also probed electrolysis of mixed lithium, potassium molten carbonates to carbon.^21^ Low carbonate melting points are achieved by a eutectic mix of alkali carbonates (*T*_mp_ Li_2_CO_3_: 723 °C, Na_2_CO_3_: 851 °C, K_2_CO_3_: 891 °C; Li_1.07_Na_0.93_CO_3_: 499 °C; Li_0.85_Na_0.61_K_0.54_CO_3_: 393 °C). We have investigated carbon dioxide and iron oxide splitting electrolysis in mixed Li, Na, K and Cs, Li, Na, K and Ba, Li carbonate electrolytes, with and without added lithium oxide, Li_2_O.^10^, ^11^, ^16^, ^22^, ^23^, ^24^, ^25^

We have delineated the solar, optical, and electronic components of STEP.^9^, ^15^, ^17^ We have previously demonstrated thermodynamically^5^, ^8^ and experimentally^6^, ^9^ that the excess heat not utilized by the concentrator photovoltaic (CPV) is sufficient to heat the electrolyzer, either alone for water splitting to H_2_, or alone for CO_2_ splitting to CO. These are combined in the illustration on the right side of Figure 1 for the simultaneous generation of H_2_ and CO from a single light source. Note that the subbandgap sunlight (IR solar heat) is energetically insufficient to drive electronic charge in the CPV. This light may be split off by a beam splitter (as demonstrated previously,^8^, ^9^ to heat the electrolyzers and lower the requisite electrolysis potential. It is not shown that incident gases (water vapor and CO_2_) are preheated prior by passing them under the heat sink below the CPV (the CPV operates more effectively at lower temperature) and that heat exchangers are used to return excess product back to the reactants prior to the electrolysis. In this study, we focus on the electrolysis component for STEP fuel to be driven electronically by the maximum power point of the CPV. Specifically, we present the first molten electrolyte sustaining electrolytic coproduction of both hydrogen and carbon monoxide from separate electrolysis with the electronic charge generated by a single beam of sunlight. The electrolysis was driven by an Envoltek 39% efficient solar‐to‐electric triple bandgap (GaInP, 1.88 eV/GaInAs, 1.41 eV/Ge, 0.67 eV) CPV as shown in **Figure** **2**. The electrolyzer current provided by an efficient CPV in lab under 1 kW Xenon, daylight color (5600 K) AM1 (air mass) illumination, the maximum power point voltage of 2.68V is used to drive the electrolysis. We introduce electrolytic conditions of CO and H_2_ production as driven by a good power match to the 2.68V maximum sustained power point of the CPV.

**Figure 2:**
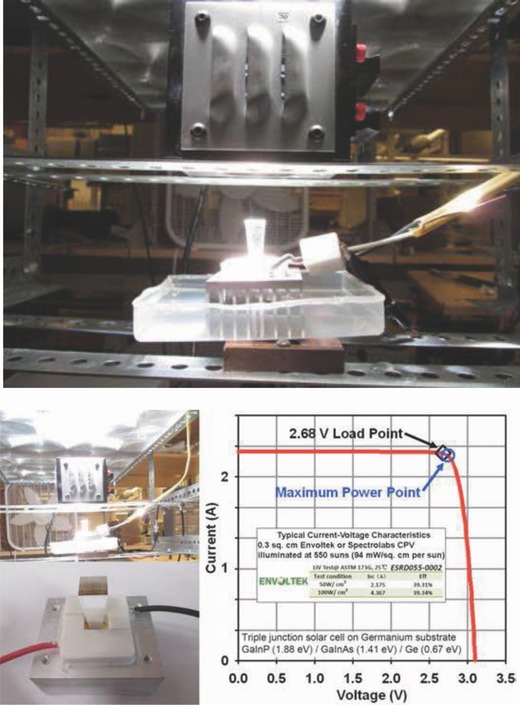
Electrolyzer current by power supply (initial experiment) and now provided by an efficient CPV in lab under 1 kW Xenon, daylight color (5600K) AM1(air mass) illumination. Left side is the 0.3074 cm^2^ Envoltek ESRD055 CPV situated under the air‐cooled AM1 filter. Middle top: the Fresnel concentrator above the AM1 filter. Middle bottom: the unattached CPV under the secondary optical concentrator. Right side: typical (550 sun) photocurrent–voltage plot of the CPV.

Previous studies show that as temperature increases, molten alkali hydroxide loses water leading to a decreasing source of water for H_2_ generation and favoring the formation of the oxide.^26^ At higher temperature, the Coulombic efficiency of H_2_ generation in molten hydroxide falls as superoxide O_2_^−^ reduction, increasingly competes with H_2_ generation in the side reactions (see the Supporting Information). In particular, we found that lithium hydroxide retains more water at higher temperature, but also only melt at higher temperatures. Whereas, LiOH melts at 462 °C and NaOH metls at 316 °C, a mixed 0.3:0.7 LiOH/NaOH eutectic of the two melts at 215 °C. We have previously demonstrated that a mixed LiOH:NaOH electrolyte retains high Coulombic efficiency of water splitting to hydrogen at elevated temperatures (99% Coulombic efficiency at 300 °C).^26^ As recounted in Table S1 of the Supporting Information, we generally observe higher Coulobmic efficiency in lithium and barium hydroxide electrolytes than in sodium or potassium hydroxide electrolytes. The mix of 0.3:0.7 LiOH/NaOH facilitates a low melting point, good water retention and the highest observed Coulombic efficiency at 300 °C. Hence, this is the electrolyte and operating temperature chosen in this study to optimize the hydrogen formation component of the light driven formation of artificial syngas (sungas).

Carbonates are ideal molten electrolytes to drive the carbon dioxide splitting reaction. Li_2_CO_3_ (723 °C, 6 S cm^−1^) has a lower melting point and higher conductivity than NaCO_3_ (851 °C, 2 S cm^−1^) and KCO_3_ (897 °C, 2 S cm^−1^).^27^ High conductivity is desired as it leads to lower electrolysis ohmic losses. One product to be generated for the solar syngas is carbon monoxide. At temperature of 950 °C in molten lithium carbonate, carbon monoxide is the preferred CO_2_ splitting product Equation (9), rather than the solid carbon product Equation (8).

The high temperature (≈950 °C) currently required to form CO in molten carbonate is a challenge to make compatible with the lower temperature range that provides a hydrated electrolyte for high yield H_2_ evolution by water splitting in the hydroxide electrolysis. Therefore, as shown in **Figure** **3**, two molten electrolysis cells, one utilizing molten LiOH/NaOH for H_2_ generation and the other molten lithium carbonate for the CO generation, were placed in series.

**Figure 3:**
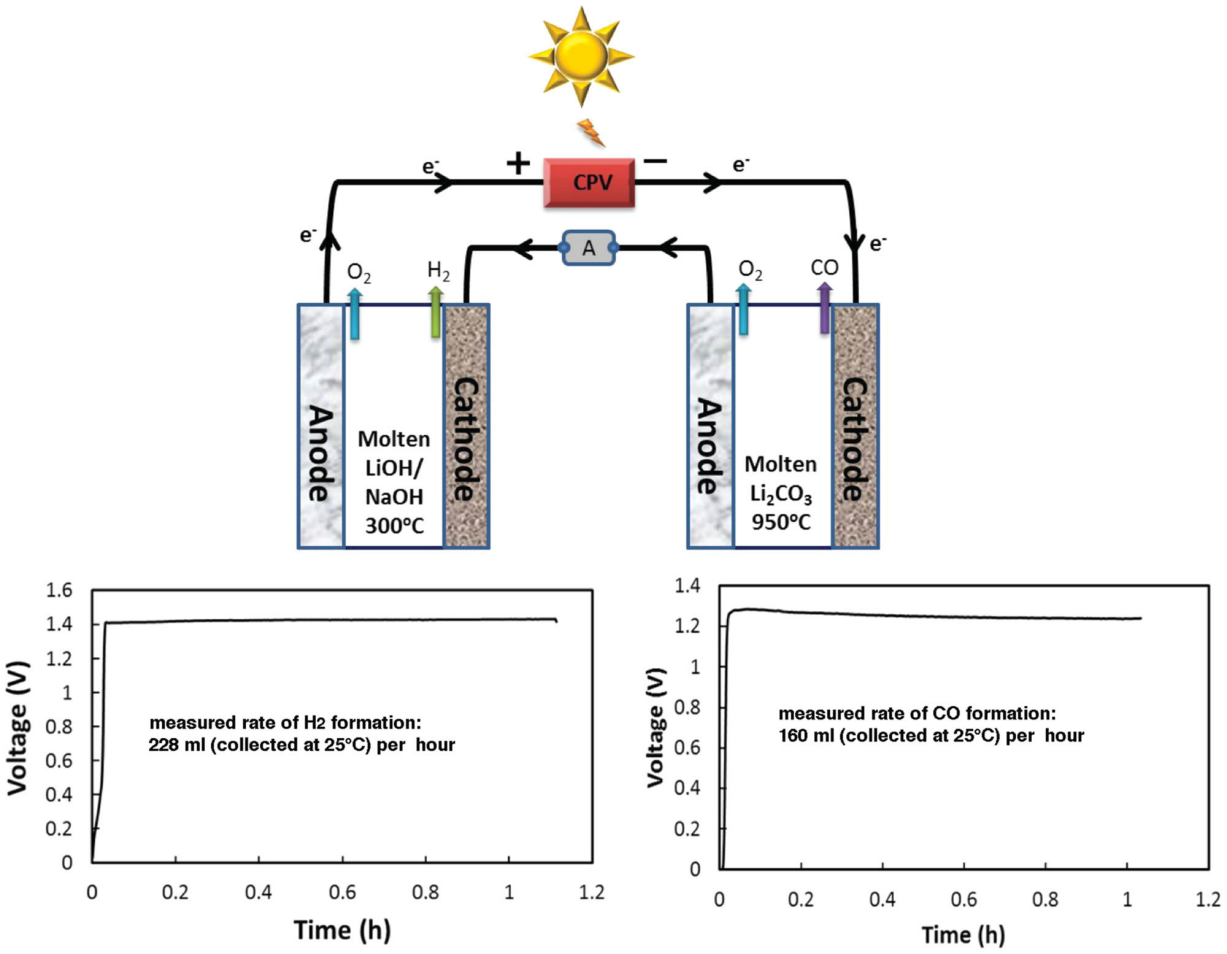
STEP fuels in which two molten electrolyzers (top left: containing a molten LiOH/NaOH electrolyte, top right: containing a molten lithium carbonate) are driven in series by a CPV. The measured electrolysis potential of water splitting to H_2_ (left) and carbon dioxide splitting to CO (right) as a function of constant current. Cathode and anode are Ni shim, each with 18 cm^2^ area.

The CPV yields a solar conversion efficiency of 38% at a maximum power point voltage of 2.68 V (Figure 2), and simultaneous in‐series electrolyses to H_2_ and CO were probed to closely match this power point voltage. 2.67 (±0.03) V is used to drive two molten electrolysis cells in series at 300 °C and 950 °C for LiOH/NaOH (3:7 molar ratio) and Li_2_CO_3_ electrolyte, respectively. The voltage curves for LiOH/NaOH and Li_2_CO_3_ electrolysis are shown in Figure 3; the average voltage for water splitting is 1.42 V, and is 1.25 V for CO_2_ splitting. The hydrogen was analyzed by both Micro IV hydrogen analyzer (GfG Instrumentation) and gas chromatograph (GC). CO was analyzed by GC. The small decrease and then leveling of the CO_2_ splitting potential during the electrolysis is attributed to an improved morphology of the aged (matte) versus fresh (smooth) cathode. The synthesis has a high Coulombic efficiency of 99% for H_2_ generating and 70% for carbon monoxide (no solid carbon product is evident at the cathode) generating at an applied electrolysis voltage (2.68V) through the 18 cm^2^ planar nickel anode and nickel cathode. Coulombic losses in the carbonate cell electrolysis can occur through recombination (reoxidation) of CO at the anode. Such losses can be prevented through a double chamber electrolysis configuration to better isolate the anode and cathode products, but can lead to higher polarization and greater cell potentials. This configuration is delineated in the Supporting Information of ref.[[qv: 18a]] (Figure S2, Supporting Information).

The simultaneous light‐driven generation of hydrogen and carbon monoxide fuels (sungas) from a single beam of light has been demonstrated at the maximum power point 39% solar conversion of a concentrator solar photovoltaic. The electrolysis electrodes and cell are durable for at least several days of operation, and both longer tests and scaled‐up (higher current) cells will be reported in future studies.

## Experimental Section

*Chemicals and Materials*: Lithium carbonate (Alfa Aesar, 99%), potassium hydroxide (Alfa Aesar, 85%). The electrolysis is contained in high‐purity alumina crucibles (99.6%, AdValue Technology AL‐2100). Electrolyses used Ni shim (Alfa Aesar, 99.5%) as both the cathode (hydrogen and carbon monoxide generating) and the (oxygen generating) anode with a Ni wire (Alfa Aesar, 99.5%) connection. Product gases are collected through a water trap, and hydrogen concentration measured using a GfG Micro IV hydrogen analyzer, and redundantly confirmed by GC, and also by the (total) collected hydrogen and oxygen volumes as previously described,^18^ and carbon monoxide concentration determined by GC (HP 5890 series II gas chromatograph). Electrolyzer current provided by an efficient CPV in lab under 1 kW Xenon, daylight color (5600K) AM1 (air mass) solar simulator illumination. Electrolysis data in this study is gathered indoors with the solar simulator. We have previously reported on the successful, outdoor‐driven two high temperature electrolyses in series mounted on a tracking heliostat outdoors using a single CPV and Fresnel heating of the electrolysis chambers.^9^, ^15^, ^17^

## Supporting information

As a service to our authors and readers, this journal provides supporting information supplied by the authors. Such materials are peer reviewed and may be re‐organized for online delivery, but are not copy‐edited or typeset. Technical support issues arising from supporting information (other than missing files) should be addressed to the authors.

Supplementary

## References

[1] K. M. K. Yu , I. Curcic , J. Gabriel , S. C. E. Tsang , ChemSusChem. 2008, 1, 893. 18985640 10.1002/cssc.200800169

[2] V. N. Nguyen , L. Blum , Chem. Ing. Tech. 2015, 87, 354.

[3] A. Andrews , J. Logan , Fisher‐Tropsch Fuels from Coal, Natural Gas, and Biomass: Background and Policy, Congressional Research Service Report for Congress 2008, Order Code RL34133, data available at http://assets.opencrs.com/rpts/RL34133_20080327.pdf(accessed September 2015)

[4] S. Licht , Electrochem. Commun. 2002, 4, 790.

[5] S. Licht , J. Phys. Chem. C 2003, 107, 4253.

[6] S. Licht , L. Halperin , M. Kalina , M. Zidman , N. Halperin , Chem. Commun. 2003, 3006. 10.1039/b309397b 14703830

[7] M. W. Chase , J. Phys. Chem. Ref. Data 1998, 9, 1, data available at http://webbook.nist.gov/chemistry/form‐ser.html

[8] S. Licht , J. Phys. Chem. C 2009, 113, 16283.

[9] S. Licht , B. Wang , S. Ghosh , H. Ayub , D. Jiang , J. Ganley , J. Phys. Chem. Lett. 2010, 1, 2363.

[10] S. Licht , Adv. Mater. 2011, 23, 5592. 22025216 10.1002/adma.201103198

[11] a) S. Licht , B. Cui , B. Wang , J. CO2 Utilization 2013, 2, 58;

[12] B. Wang , H. Wu , G. Zhang , S. Licht , ChemSusChem 2012, 5, 2000. 22965739 10.1002/cssc.201200305

[13] B. Wang , Y. Hu , H. Wu , S. Licht , Electrochem. Sci. Lett. 2013, 2, H34.

[14] a) Y. Zhu , B. Wang , X. Liu , H. Wang , H. Wu , S. Licht , Green Chem. 2014, 16, 881;

[15] a) S. Licht , H. Wu , J. Phys. Chem. C 2011, 115, 25138;

[16] B. Cui , S. Licht , Green Chem. 2013, 15, 881.

[17] S. Licht , H. Wu , C. Hettige , B. Wang , J. Lau , J. Asercion , J. Stuart , Chem. Commun. 2012, 48, 6019. 10.1039/c2cc31341c 22540130

[18] a) S. Licht , B. Cui , B. Wang , F.‐F. Li , J. Lau , S. Liu , Science 2014, 345, 637; 25104378 10.1126/science.1254234

[19] S. Licht , O. Chityat , H. Bergmann , A. Dick , S. Ghosh , H. Ayub , Int. J. Hyd. Energy 2010, 35, 10867.

[20] V. Kaplan , E. Wachtel , K. Gartsman , Y. Feldman , I. Lubomirsky , J. Electrochem. Soc. 2010, 157, B552.

[21] H. Ijije , C. Sun , G. Chen , Carbon 2014, 73, 163.

[22] B. Cui , S. Licht , J. Mat. Chem. A 2014, 2, 10577.

[23] R. I. Olivares , C. Chen , S. Wright , J. Solar Energy Eng. 2012, 134, 41002.

[24] http://www.crct.polymtl.ca/fact/documentation./FTsalt/FTsalt_Figs.htm (accessed September 2015)

[25] S. Licht , **2012**, arXiv:1209.3512.

[26] F.‐F. Li , S. Liu , B Cui , J. Lau , J. Stuart , B. Wang , S. Licht , Adv. Energy Mater. 2015, 5, 1401791.

[27] Principles and Applications of Molten Salt Electrochemistry (Eds: Zhang Z., Wang Z.), Chemical Industry Press, Beijing, P.R. China 2006, p. 191.

