# Supplementary material for: Sungas Instead of Syngas: Efficient Coproduction of CO and H2 with a Single Beam of Sunlight
Source: Adv Sci (Weinh). 2015 Oct 1;2(11):1500260. doi: 10.1002/advs.201500260 (PMC5054927; doi:10.1002/advs.201500260)
Supplement: Supplementary file 1 — Supplementary [file ADVS-2-0a-s001.pdf]

Copyright WILEY-VCH Verlag GmbH & Co. KGaA, 69469 Weinheim, Germany, 2015.

ADVANCED SCIENCE
Open Access

Supporting Information

for *Adv. Sci.*, DOI: 10.1002/advs. 201500260

Sungas Instead of Syngas: Efficient Coproduction of CO and H_2 with a Single Beam of Sunlight

*Fang-Fang Li , Jason Lau , and Stuart Licht **

Copyright WILEY-VCH Verlag GmbH & Co. KGaA, 69469 Weinheim, Germany, 2013.

Supporting Information

Sungas instead of syngas:
Efficient co-production of CO and H_2 with a single beam of sunlight

*Fang-Fang Li, Jason Lau, and Stuart Licht**

Syngas is commonly produced to convert solid or gas-phase fossil fuels into liquid fuels.[2] The conventional steam reformation production of syngas from methane is realized by steam reforming reactions to $CO + H_2$, given by eqs 1-3:
by steam reforming reactions to $CO + H_2$, given by eqs 1-3:

$$CH_4 + H_2O \rightarrow CO + 3H_2 \quad (1)$$
$$CH_4 + CO_2 \rightarrow 2CO + 2H_2 \quad (2)$$
$$CH_4 + 1/2O_2 \rightarrow CO + 2H_2 \quad (3)$$

and the production of syngas from coal is realized by:

$$3C + O_2 + H_2O \rightarrow 3CO + H_2 \quad (4)$$

The water shift reaction is then used if more hydrogen is desired from the coal gas product:

$$CO + H_2O \rightarrow CO_2 + H_2 \quad (5)$$

The syngas is then used as a feedstock to form methanol, or for Fischer-Tropsch type reactions to produce middle-distillate range fuels of C11-C18 hydrocarbons including synthetic jet, kerosene and diesel fuels.[3]

Low temperature enhances electrolytic H_2 formation in molten hydroxides. The coulombic efficiency of electrolytic water splitting, η_{H_2}, (moles H_2 generated per 2 Faraday of applied charge) approaches 100% in low melting point, mixed alkali molten hydroxides at temperatures up to 300°C.

$$H_2O \rightarrow H_2 + 1/2O_2 \quad (1)$$
$$\text{cathode:}\ 2H_2O + 2e^- \rightarrow H_2 + 2OH^- \quad (2)$$
$$\text{anode:}\ 2OH^- \rightarrow 1/2O_2 + H_2O + 2e^- \quad (3)$$

Hydroxide hydrates, $MOH\cdot nH_2O$ (M=alkali), bind fewer waters, n, at higher temperature, equilibria, and saturated aqueous alkali hydroxide solutions contain less water. For example, saturated NaOH solution equilibrate with 23%, 16% or 3% water respectively at 100°C, 200°C or 300°C (saturated aqueous NaOH solution do not boil at 1 atmosphere of pressure[3]), As temperature increases, molten alkali hydroxides provide a decreasing source of water for hydrogen generation as they lose water favoring the formation of the oxide:

$$2MOH \rightarrow M_2O + H_2O(gas) \qquad (4)$$

For example, as temperature increases by 100°C increments from 500°C to 900°C, the equilibria constant for the LiOH reaction of eq 5 (M=Li), increases from 0.006, 0.02, 0.1, 0.3, to 0.8. [23] At higher temperature the coulombic efficiency of hydrogen generation in molten hydroxide falls as superoxide, O_2^- reduction, increasingly competes with hydrogen formation in the (parasitic) side reactions:[19]

$$\text{anode: } 4OH^- \rightarrow O_2^- + 2H_2O + 3e^- \qquad (5)$$

$$\text{cathode: } O_2^- + 2H_2O + 3e^- \rightarrow 4OH^- \qquad (6)$$

Table S1. The measured coulombic efficiency of hydrogen generation, η_{H_2}, in various molten hydroxide electrolytes at various temperatures. Water (as steam) is bubbled through the electrolyte. Electrolytic water splitting is measured at 267 mA cm^{-2} (1 A) between two planar 3.75 cm^2 nickel electrodes. Efficiency is determined by comparing the measured mole equivalents of hydrogen generated to each 2 mole equivalents (coloumbs) of applied current in accord with $H_2O \rightarrow H_2 + 1/2O_2$.

Electrolyte	Melting Point	η_{H_2}(200°C)	η_{H_2}(300°C)	η_{H_2}(400°C)	η_{H_2}(500°C)	η_{H_2}(600°C)	η_{H_2}(700°C)	η_{H_2}(800°C)
LiOH	462°C				88%	21%	4%	0%
$Li_{0.3}Na_{0.7}OH$	215°C		99%	87%	47%			
NaOH	318°C			72%				
$Na_{0.5}K_{0.5}OH$	170°C	96%	86%	47%	28%	13.4%		
KOH	406°C			80%				
$Li_{0.3}K_{0.7}OH$	225°C		94%	56%	32%	7.8%		
$Ba(OH)_2$	407°C				55%	32%		
$Li_{0.33}Ba_{0.67}(OH)_2$			96%					
$(NaOH)_{0.865}(Na_2CO_3)_{0.125}$				77%				
$(LiOH)_{0.843}(Li_2CO_3)_{0.157}$					79%	17.2%	5.5%	
